# Patient classification of hypertension in Traditional Chinese Medicine using multi-label learning techniques

**DOI:** 10.1186/1755-8794-8-S3-S4

**Published:** 2015-09-23

**Authors:** Guo-Zheng Li, Zehui He, Feng-Feng Shao, Ai-Hua Ou, Xiao-Zhong Lin

**Affiliations:** 1 Department of Big Medical Data, Guangdong Provincial Hospital of Chinese Medicine, The Second Affiliated Hospital of Guangzhou University of Chinese Medicine, Guangzhou, China; 2 Department of Control Science and Engineering, Tongji University, Shanghai, China; 3 Department of Cardiology, Guangdong Provincial Hospital of Chinese Medicine, The Second Affiliated Hospital of Guangzhou University of Chinese Medicine, Guangzhou, China

**Keywords:** Traditional Chinese medicine, Multi-label learning, Data mining, Feature selection

## Abstract

**Background:**

Hypertension is one of the major risk factors for cardiovascular diseases. Research on the patient classification of hypertension has become an important topic because Traditional Chinese Medicine lies primarily in "treatment based on syndromes differentiation of the patients".

**Methods:**

Clinical data of hypertension was collected with 12 syndromes and 129 symptoms including inspection, tongue, inquiry, and palpation symptoms. Syndromes differentiation was modeled as a patient classification problem in the field of data mining, and a new multi-label learning model BrSmoteSvm was built dealing with the class-imbalanced of the dataset.

**Results:**

The experiments showed that the BrSmoteSvm had a better results comparing to other multi-label classifiers in the evaluation criteria of Average precision, Coverage, One-error, Ranking loss.

**Conclusions:**

BrSmoteSvm can model the hypertension's syndromes differentiation better considering the imbalanced problem.

## Background

Hypertension is one of the major risk factors of cardiovascular diseases. It contributes to one half of the coronary heart disease and approximately two thirds of the cerebrovascular disease burdens [1]. There are over 972 million hypertension patients in the world [2].Traditional Chinese Medicine (TCM) has been playing an important role on treating hypertension, and it lies primarily in "treatment based on syndrome differentiation of the patients". Traditionally, syndrome differentiation is performed by TCM practitioner should have solid theoretical foundation and plentiful experiences.

In the field of data mining, syndrome differentiation can be regarded as a patient classification problem which can be solved with specific data mining and machine learning techniques. It has become a fast developing field with the accumulating of clinical data [3-6].

In traditional classification problems, one case would be only classified to one category (i.e. label) which is called single label classification. While in TCM, one patient may have more than one syndromes which should be multi-label classification problems in the data mining field. Multi-label learning has been used in TCM field and got better results comparing with conventional learning methods. Liu et al. compared the performance of Multi-label-KNN and KNN on a coronary heart disease dataset. Li et al. had investigated the contribution of symptoms to syndromes diagnosis by using fusion symptoms with ML-KNN classifier [7]. Li et al. and Shao et al. proposed embedded multi-label feature selection method MEFS [8] and wrapper multi-label feature selection method HOML [9], respectively, to get better performance for the multi-label classification.

Multi-label classification was mainly motivated by the tasks of text categorization and medical diagnosis in the past. The existing methods for multi-label classification can be grouped into two main categories: a) problem transformation methods, and b) algorithm adaptation methods. The problem transformation methods transform the multi-label classification problem either into one or more single-label classification or regression problems, and there have been many learning algorithms depending on transformation methods. The algorithm adaptation methods could extend specific learning algorithms to deal with multi-label data directly [10].

In classification, a dataset is said to be imbalanced when the number of cases which represents one class is much smaller than the ones from other classes [11]. Furthermore, the class with the lowest number of cases is usually the class of interest from the point of view of the learning task [12]. This phenomenon is of great interest as it turns up in many real-world classification problems, such as risk management [13], fraud detection [14], and especially medical diagnosis [15-19].

In this study, a new classification method named BrSmoteSvm is built for hypertension syndromes differentiation. The BrSmoteSvm works on both multi-label data and class-imbalanced problem. It is a combination of Binary Relevance (BR), Synthetic Minority Over-sampling Technique (SMOTE) [16] and Support Vector Machine (SVM) [17]. Firstly, BR algorithm is used to transform the multi-label classification problem into single-label classification. And it is found class-imbalance on the single-label situation. Then, SMOTE is applied to decrease the effect of the class-imbalance problem. At last, SVM is used as the binary classifier to differentiate the syndromes.

The rest of this paper is arranged as follows. Section 2 describes the materials and the methods of this study. Section 3 presents the results and discussion of our experiment. Section 4 presents the conclusions.

## Methods

### Materials

The study dataset originated from the hypertension patients who visited the in-patient and out-patient departments of Internal Medicine, Nerve Internal Medicine and Health Management Center of the Guangdong Provincial Hospital of Chinese Medicine and Li Wan District Community Hospital in Guangzhou of China during November 2006 to December 2008. This study was approved by the ethics committee of the Guangdong Provincial Hospital of Chinese Medicine, China. Informed written consent was obtained from each participant prior to data collection. In total, 908 cases were collected with 13 syndromes and 129 TCM symptoms from inspection symptoms, tongue symptoms, inquiry symptoms, palpation symptoms and other symptoms.

Four cases were excluded from the analysis because of missing answers on features. And one syndrome were excluded because of its nonnumeric value to make sure the smooth application of data mining methods. Finally, we got 904 cases with 12 syndromes and 129 symptoms. Table 1 shows the number of cases (D); the number of features (M); the number of labels (|L|); the Label Cardinality (LC), which is the average number of single-labels associated with each example defined by $\text{LC}\left(\text{D}\right)=\frac{1}{\left|D\right|}\sum_{i=1}^{\left|D\right|}|Y_{i}|$; the Label Density (LD), which is the normalized cardinality defined by $\text{LC}\left(\text{D}\right)=\frac{1}{\left|D\right|}\sum_{i=1}^{\left|D\right|}\frac{\left|Y_{i}\right|}{L},L={⋂}_{i=1}^{\left|D\right|}Y_{i}$; the number of Distinct Combinations (DC) of labels. |D| represents the number of examples and |Y_i_| represents the label number of the *i *case.

**Table 1:** Description of the datasets.

Dataset	Domain	N	M	|L|	LC	LD	DC
hypertension	medical	904	129	12	0.86	0.07	57

### Computational methods

In multi-label classification, each case could have several syndromes. The cases are associated with a subset of labels Y⊆*L *where *L *is the set of possible labels. Following is a brief introduction of the algorithms used in this study.

1) SMOTE

SMOTE is used to decease the influence of the class-imbalanced problem. It is an over-sampling approach in which the minority class is over-sampled by creating "synthetic" examples. The main idea of SMOTE can be described as follows.

Step 1: Compute the k nearest neighbors for each minority class instance. Randomly choose N of the k nearest neighbors of each minority class instance saved as Populate.

Step 2: Take the difference of the feature vector between each minority class instance and its nearest neighbors in Populate. Multiply this difference by a random number between 0 and 1, and add it to the feature vector of each minority class instance.

The synthetic examples generated by SMOTE cause the classifier to create larger and less specific decision regions rather than smaller and more specific regions. More general regions are now learned for the minority class samples rather than those being subsumed by the majority class samples around them.

2) SVM

SVM is used as the binary classifier in BR. The original SVM algorithm was invented by Vladimir N. Vapnik and the current standard incarnation (soft margin) was proposed by Vapnik and Corinna Cortes in 1995. The basic SVM takes a set of input data and related label, and for each given input, two possible classes forms the output, making it a non-probabilistic binary linear classifier. Given a set of training instances, each marked as belonging to one of two classes, an SVM training algorithm builds a model that can assign new instances into one class or the other. An SVM model is a representation of the instances as points in space, mapped so that the instances of the separate classes are divided by a clear gap and the gap is as wide as possible. The test instances are then mapped into that same space and predicted to belong to a class based on which side of the gap they fall on. The above describes that SVM performs a linear classification. In addition, SVM can also efficiently perform a non-linear classification using what is called the kernel trick, implicitly mapping their inputs into high-dimensional feature spaces.

3) BrSmoteSvm

The main idea of BrSmoteSvm is described as follows. In each fold of the 10-fold cross validation, BR, a problem transformation method is used. The basic idea of BR is to decompose the multi-label learning problem into q independent binary classification problems, where q is the number of label and each binary classification problem corresponds to a possible label in the label space [18]. Therefore, for any multi-label training example, each instance will be involved in the learning process of q binary classifiers. Then SMOTE is applied to training data to decrease effect of the class-imbalanced problem. In the end, SVM is used as the binary classifier. After the 10-fold cross validation, we get the predicted label set.

#### Experimental design and evaluation

In our experiment, 10-fold cross validation is utilized to test the accuracy of the classification. Let 700 cases of the data be training set, and 204 cases be testing set. In order to validate performance of BrSmoteSvm, it is compared with other popular multi-label classifiers.

1) ML-KNN. The number of neighbors is set to 10 and the smoothing factor is set to 1 as recommended.

2) Random k-Labelsets (RAKEL) [19]. J48 is used as the base learner; the number of models is set to 5; the size of subsets is set to 8.

3) Instance-based learning and logistic regression (IBLR) [20]. The number of nearest neighbors is set to 10.

4) Ensemble of Classifier Chains (ECC). J48 is used as the base learner for each Classifier Chains model; the number of models is set to 10.

5) A lazy multi-label classification method based on the KNN (BRKNN) [21]. The number of the nearest neighbors is set to 10.

At last, for SMOTE, N is set to fixed value 10, and k is chosen from {10, 12, 14, 16, 18, and 20}; then, k is set to fixed value 16, and N is chosen from {6, 8, 10, 12, 14, and 16} to evaluate the robustness of our method.

Let × denote the domain of cases and let *Y*={1,2,...,Q} be the set of labels. The purpose of the learning system is to output a multi-label classifier h: X→2y for the given training set through optimizing some specific evaluation metric. In other word, a successful learning system would output larger values for labels in *Y_i _*than those not in *Y_i _*for the given instance xi and its label set *Y_i_*. For example, *f *(*x_i_,y_i_*)>*f *(*x_i_,y_j_*) for any *y_i _*in *Y_i _*and *y_j _*not in *Y_i_*.

The real-value function *f *(.,.) can be transformed to a ranking function *rank*(.,.), which maps the outputs of *f*(*x_i_,y*) for any y in *Y *to {1,2,...,Q} such that if *f *(*x_i_,y_i_*) >*f *(*x_i_,y_j_*) then *rank*(*x_i_,y_i_*) <*rank*(*x_i_,y_j_*). For a test set *S={(x_1_,Y_1_),(x_2_,Y_2_),...,(x_p_,Y_p_)}*, the following criteria are used in this study:

1) Hamming loss: defined as:

$$\text{hamming loss}\left(h\right)\text{ = }\frac{1}{p}\overset{p}{\underset{i=1}{\sum }}\frac{1}{Q}\left|h\left(x_{i}\right)\Delta Y_{i}\right|$$

where Δ stands for the symmetric difference between two sets. Note that when | Yi | = 1 for all instances, a multi-label system is in fact a multi-class single-label one and the hamming loss is 2/Q times the usual classification error. Hamming loss is used to evaluate how many times an instance-label pair is misclassified. The smaller the value of Hamming loss (h), the better the performance.

2) One-error: defined as:

$$\text{one error}\left(f\right)=\frac{1}{p}\overset{p}{\underset{i=1}{\sum }}\left[\left[\text{arg}\underset{y\in Y}{\text{max}}f\left(x_{i},y\right)\right]\notin Y_{i}\right].$$

Note that, for single-label classification problems, the one-error is identical to ordinary classification error. One-error is used to evaluate how many times the top-ranked label is not in the set of proper labels of the instance. The smaller the value of one-error (*f*), the better the performance.

3) Coverage: defined as:

$$\text{coverage}\left(f\right)=\frac{1}{p}\overset{p}{\underset{i=1}{\sum }}\underset{y\in Y_{i}}{\text{max}}rank_{f}\left(x_{i},y\right)-1,$$

evaluates how far we need, on the average, to go down the list of labels in order to cover all the proper labels of the instance. It is loosely related to precision at the level of perfect recall. The smaller the value of coverage (*f*), the better the performance.

4) Ranking loss: defined as:

$$\text{ranking loss}\left(f\right)=\frac{1}{p}\overset{p}{\underset{i=1}{\sum }}\frac{\left|D\right|}{\left|Y_{i}\right|\left|{\bar{Y}}_{i}\right|},$$

$$D=\left\{\left(y_{1},y_{2}\right)f\left(x_{i},y_{1}\right)\leq f\left(x_{i},y_{2}\right),\;\;\left(y_{1},y_{2}\right)\in Y_{i}\times \bar{Y_{i}}\right\},$$

where $\bar{Y}$ denotes the complementary set of *Y *in *y*. Ranking loss is used to evaluate the average fraction of label pairs that are reversely ordered for the instance. The smaller the value of ranking loss (*f*), the better the performance.

5) Average precision: defined as:

$$average\;precision\left(f\right)=\frac{1}{p}\overset{p}{\underset{i=1}{\sum }}\frac{1}{\left|Y_{i}\right|}\underset{y\in Y_{i}}{\sum }\frac{\left|L_{i}\right|}{rank_{f}\left(x_{i},y\right)},$$

$$L_{i}=\left\{y^{\prime }|rank_{f}\left(x_{i},y^{\prime }\right)\leq rank_{f}\left(x_{i},y\right),y^{\prime }\in Y_{i}\right\},$$

which is used to evaluate the average fraction of labels ranked above a particular label *y*∈*Y *which actually are in *Y*. The bigger the value of *average precision *(*f*), the better the performance.

## Results and discussion

### Comparison with other multi-label classifiers

The 10-fold cross validation was applied to test the accuracy of classification in which BrSmoteSvm was compared with other five multi-label classifiers. Results of 10-fold cross validation are shown in Table 2. In Table 2, the Average precision of BrSmoteSvm is 0.66, which is much higher than the results of other methods. For Coverage, One-error and Ranking loss, BrSmoteSvm also performs better than other methods. While, for Hamming loss, BrSmoteSvm is 0.09, which performs worse than other methods.

**Table 2:** Results of BrSmoteSvm and other multi-label classifiers using 10-fold cross validation.

	BrSmoteSvm	MLKNN	BRKNN	ECC	IBLR	RAKEL
Average precision	0.66	0.53	0.51	0.51	0.51	0.46
Hamming loss	0.09	0.07	0.07	0.07	0.07	0.09
Coverage	1.11	2.21	2.46	2.41	2.34	2.89
One-error	0.47	0.75	0.75	0.76	0.76	0.78
Ranking loss	0.16	0.16	0.18	0.18	0.17	0.22

The reason of the large number of Hamming loss might be serious imbalance of the dataset. For most labels, there are only 20 to 70 positive cases, which means the ratio of the negative and positive cases is very high. On the other side, for the low number of the positive case, the classifier would be trained insufficiently producing bad performance of the testing data. Performance of machine learning methods is typically evaluated using predictive accuracy. It would be inappropriate when the data set is imbalance or the cost of different errors vary significantly. So, the simple predictive accuracy is inappropriate in this situation. In this study, SMOTE is applied to decrease the effect of the imbalance problem. The rate of detection positive cases would be improved, while the error rate for the negative cases be increased.

Another reasons could be that SMOTE might not be the best method dealing with the imbalance of the dataset, and the parameters for the algorithms used were not optimal. Further studies could focus on how to deal with the imbalanced problem and optimize the algorithms.

Furthermore, an experiment was conducted to compare the results with SMOTE and without SMOTE. The results are shown in Table 3. BrSmoteSvm+SMOTE represents with SMOTE, and BrSmoteSvm-SMOTE represents without SMOTE. It shows that the results with SMOTE are better than the results without SMOTE.

**Table 3:** Results of BrSmoteSvm with and without SMOTE.

	BrSmoteSvm+SMOTE	BrSmoteSvm-SMOTE
Average precision	0.66	0.58
Hamming loss	0.09	0.07
Coverage	1.11	1.36
One-error	0.47	0.59
Ranking loss	0.16	0.19

### Stability of BrSmoteSvm

**Figure 1:**
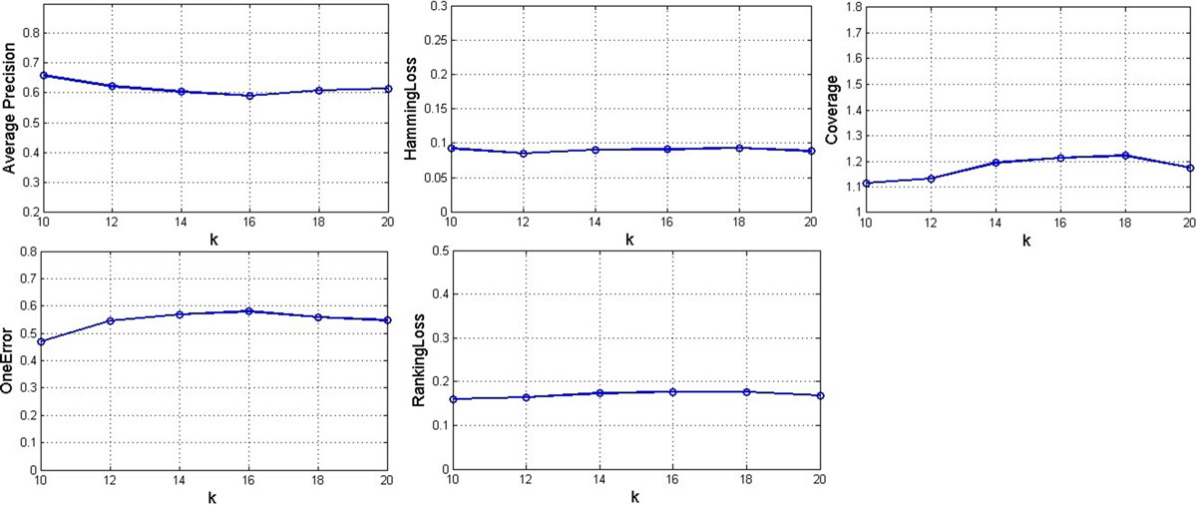
**Results of BrSmoteSvm with different k values and fixed N value for SMOTE**.

**Figure 2:**
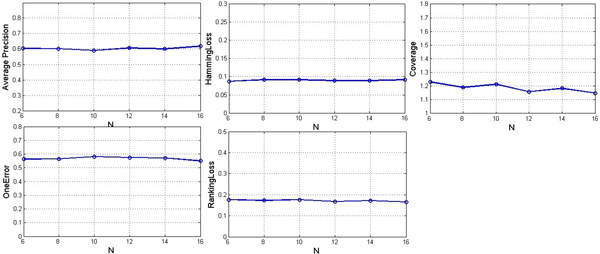
**Results of BrSmoteSvm with fixed k value and different N values for SMOTE**.

Two experiments were designed to validate the stability of BrSmoteSvm. The first one set N fixed as 10, and k was from {10, 12, 14, 16, 18, and 20} for SMOTE. The second one set k fixed as 16, and N was from {6, 8, 10, 12, 14, and 16}. The results of the two experiments are shown in Figure 1 and 2 using the evaluation criteria of Average precision, Hamming loss, Coverage, One-error and Ranking loss.

Figure 1 and 2 illustrate that:

1) The results of BrSmoteSvm vary with different k and N, but the change is small, indicating BrSmoteSvm is stable.

2) Whatever k and N values, BrSmoteSvm performs better than other methods in the evaluation of Average precision, Coverage, One-error and Ranking loss except for Hamming loss.

3) When k and N are both set to 10, BrSmoteSvm obtains the best performance.

## Conclusions

Pattern classification is important in TCM for specific disease like hypertension. However, there are multi-labels of syndromes in patients, and the numbers of patients under each syndromes are so skew that classification performance is reduced. BrSmoteSvm is proposed by combining multi-label learning and SMOTE, to help overcome the effects of multi-labels and skew numbers of patients of syndromes. Results of experiments showed that BrSmoteSvm improves the performance of the previous works. Multi-label learning and imbalance learning techniques are necessary to process the medical data sets with above problems.

Further work may focus on novel combination of multi-label learning and imbalance learning techniques to improve the accuracy of classification.

## Abbreviations used

BR: Binary Relevance

SMOTE: Synthetic Minority Over-sampling Technique

SVM: Support Vector Machine

## Competing interests

The authors declare that they have no competing interests.

## Authors' contributions

GZL and ZH contributed to the design of the study, the critical revision of the manuscript. FFS performed the statistical analysis and drafted the manuscript. AHO and XZL planned and monitored the data collection procedures. All authors read and approved the final manuscript.
